# Integrated Analysis of Tissue-Specific Promoter Methylation and Gene Expression Profile in Complex Diseases

**DOI:** 10.3390/ijms21145056

**Published:** 2020-07-17

**Authors:** Kibaick Lee, Sanghoon Moon, Mi-Jin Park, In-Uk Koh, Nak-Hyeon Choi, Ho-Yeong Yu, Young Jin Kim, Jinhwa Kong, Hee Gyung Kang, Song Cheol Kim, Bong-Jo Kim

**Affiliations:** 1 Division of Genome Research, Center for Genome Science, Korea National Institute of Health, Chungcheongbuk-do 28519, Korea; kibaicklee@korea.kr (K.L.); moon.sanghoon@daum.net (S.M.); pmjbibi@korea.kr (M.-J.P.); kohinuk@korea.kr (I.-U.K.); knihep@korea.kr (N.-H.C.); hoyeong6428@korea.kr (H.-Y.Y.); inthistime@korea.kr (Y.J.K.); kjh594@korea.kr (J.K.); 2 Department of Pediatrics, Seoul National University College of Medicine, Seoul 03080, Korea; kanghg@snu.ac.kr; 3 Department of Surgery, Asan Medical Center, AMIST, University of Ulsan College of Medicine, Seoul 05505, Korea

**Keywords:** tissue-specific expression, DNA methylation, type 2 diabetes, obesity

## Abstract

This study investigated whether the promoter region of DNA methylation positively or negatively regulates tissue-specific genes (TSGs) and if it correlates with disease pathophysiology. We assessed tissue specificity metrics in five human tissues, using sequencing-based approaches, including 52 whole genome bisulfite sequencing (WGBS), 52 RNA-seq, and 144 chromatin immunoprecipitation sequencing (ChIP-seq) data. A correlation analysis was performed between the gene expression and DNA methylation levels of the TSG promoter region. The TSG enrichment analyses were conducted in the gene–disease association network (DisGeNET). The epigenomic association analyses of CpGs in enriched TSG promoters were performed using 1986 Infinium MethylationEPIC array data. A correlation analysis showed significant associations between the promoter methylation and 449 TSGs’ expression. A disease enrichment analysis showed that diabetes- and obesity-related diseases were high-ranked. In an epigenomic association analysis based on obesity, 62 CpGs showed statistical significance. Among them, three obesity-related CpGs were newly identified and replicated with statistical significance in independent data. In particular, a CpG (cg17075888 of *PDK4*), considered as potential therapeutic targets, were associated with complex diseases, including obesity and type 2 diabetes. The methylation changes in a substantial number of the TSG promoters showed a significant association with metabolic diseases. Collectively, our findings provided strong evidence of the relationship between tissue-specific patterns of epigenetic changes and metabolic diseases.

## 1. Introduction

The human body consists of more than 200 types of cells in various tissues that perform specific functions in different biological processes [1]. Tissue-specific genes (TSGs) are specifically expressed in their respective tissues, in contrast to housekeeping genes. The discovery of TSGs has broadened our understanding of the functions of various tissues [2,3,4], as well as their biological mechanisms [5], since the aberrant expression of TSGs may be implicated in various diseases [6].

Epigenetic events, including DNA methylation and histone modification, are key regulators of gene expression and phenotype [7]. Methylation changes in the promoter region generally act as silencers of downstream genes [8,9,10]. Reportedly, gene body methylation positively regulates gene expression [11,12,13]. Since the epigenetic level differs across human tissues, it is important to explore epigenetic factors to understand the mechanisms underlying TSGs.

To date, several studies have been conducted to examine the relationship between gene expression and DNA methylation. However, most of these studies had limitations in investigating the relationship between the epigenetic changes and gene expression levels. First, since array-based studies relied on predefined markers and/or hybridization efficiencies, their resolution was not sufficient to cover the entire genome. A systematic TSG analysis by massive mRNA-sequencing [14] would be required for this purpose. Second, cohort studies mainly used DNA samples from peripheral blood. Although blood DNA is an excellent material for epigenetic studies, and biological phenomena in the blood environment closely reflect those in target cells or tissues, explaining tissue specificity using only blood DNA remains difficult. Finally, previous studies focused on either a single target (single gene or single tissue) or performed case-control analyses. However, it remained challenging to investigate the correlations between DNA methylation and gene expression based on the inter-tissue and inter-individual differences. Most recently, Blake and colleagues applied a systematic analysis to gene expression and DNA methylation patterns in multiple tissues across species, utilizing a genome sequencing-based profiling to overcome the above-mentioned limitations [15]. Moreover, some functional studies were conducted on this subject [15,16,17].

In the present study, we performed an integrated analysis of matched samples, employing whole genome bisulfite sequencing (WGBS), mRNA-seq, and ChIP-seq (chromatin immunoprecipitation followed by sequencing), to investigate the relationship between the methylation of the promoter region and expression of the gene, based on tissue specificity. We also analyzed the gene expression pattern influenced by epigenetic regulation, according to the direction of correlation (positive or negative), and investigated the relevance of the disease by a network analysis and epigenetic association analysis. Consequently, we identified metabolic disease-associated CpG markers within the promoter of TSGs, involved in controlling gene expression in practice.

## 2. Results

### 2.1. Identification of TSGs by Gene Expression Patterns

All mRNA-seq data were uniformly pre-processed by the International Human Epigenome Consortium (IHEC) standard mRNA-seq pipeline (see Materials and Methods). In brief, all FASTQ files were mapped to human genome assembly GRCh37, and read counts of each gene were counted based on the GENCODE v19 gene model. A downstream analysis was performed on 11,111 protein-coding genes. We then profiled gene expression. We detected no batch effect in any group (Figure S1). Next, a pairwise correlation analysis was performed based on the transcripts per million (TPM) value. While samples from the same tissue were highly correlated, those from different tissues showed weaker correlations (mean Pearson’s correlation coefficients were 0.956 and 0.721, respectively; Figure S2).

**Figure 1:**
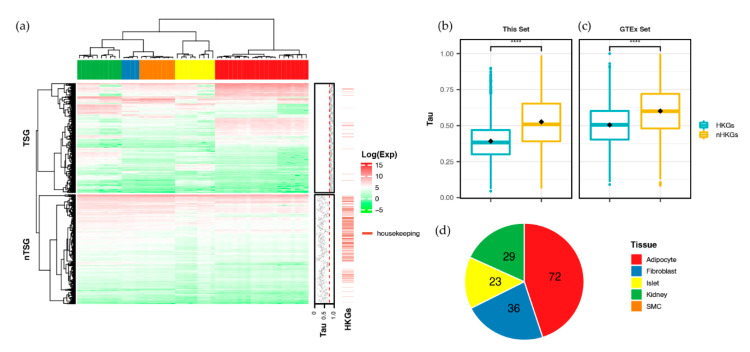
Gene expression revealed a tissue-specific pattern. (**a**) The gene expression pattern of protein-coding genes. (**b**,**c**) The distribution of Tau revealed whether the gene is housekeeping or not: our set (**b**) and the Genotype-Tissue Expression (GTEx) set (**c**). (**d**) A summary of all the common tissue-specific genes (TSGs) in the two data sets. nTSG, not TSG; HKGs, housekeeping genes; nHKGs, non-housekeeping genes.

We identified 677 TSGs, with Tau ≥ 0.8 (threshold (the number of TSGs for adipocyte, fibroblast, islet, kidney, and skeletal muscle cells was 248, 62, 89, 241, and 37, respectively; Table S1)). Each TSG was highly expressed in specific tissues, while 677 non-specific genes, which were randomly sampled with Tau < 0.8, showed a ubiquitous expression in all tissues (Figure 1a). We re-analyzed the expression data from the Genotype-Tissue Expression (GTEx) version 7 [18], using the same metric to confirm tissue specificity. The distribution of Tau for both housekeeping [19] and non-housekeeping genes was comparable in both sets (Figure 1b,c). The number of genes common with the TSGs in the GTEx set was 160; the total number of genes is shown in Figure 1d.

### 2.2. Selection of Promoter Region Based on Methylation Levels

We assessed the methylation patterns of the promoter region for 11,111 protein-coding genes. The methylation level within the proximal promoter was profiled instead of that within the typical promoter (region from the transcription start site (TSS) to 2 kb upstream) due to its functional and regulative importance in transcriptional processes [20]. Furthermore, there are many transcription factor binding sites (TFBS) in the proximal promoter regions that are responsible for transcriptional regulation [21]; therefore, this region may have open chromatin and result in a low methylation level. We calculated the whole genome-scale methylation level of a single-base pair resolution using Python script in the whole genome bisulfite sequence MAPping program (BSMAP), and then extracted the methylation level of the promoter region based on the gene model of GENCODE v19. We obtained the average of all the promoter methylation levels. Our results revealed that the methylation level of proximal promoters was lower than that of a typical promoter. The mean methylation level of proximal and typical promoters was 9.3% and 23%, respectively (Figure S3).

### 2.3. Correlation Analysis Between Gene Expression and Promoter Methylation

To identify the relationship between gene expression and promoter methylation based on tissue specificity, we performed a correlation analysis of each gene. The correlation analysis in five tissues showed 449 significant expression–methylation correlations at the threshold (with an absolute value of Pearson’s correlation coefficient ≥ 0.3) out of a total of 677 TSGs. The number of genes that negatively and positively correlated are 337 and 112, respectively (Table 1).

**Figure 2:**
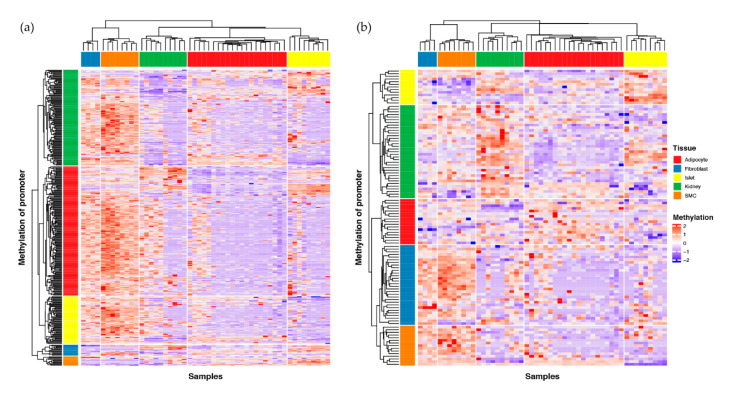
The proximal promoter methylation pattern according to the correlation between the gene expression and methylation levels. (**a**) The proximal promoter methylation patterns of 337 genes that were negatively correlated with gene expression (τ > 0.8, Pearson’s correlation coefficient < −0.3). (**b**) The proximal promoter methylation patterns of 112 genes that correlated positively with gene expression (τ > 0.8, Pearson’s correlation coefficient > 0.3).

For the 337 negatively correlated genes, we performed a supervised hierarchical clustering analysis based on the methylation pattern of the proximal promoter region. We first divided the samples and methylation levels of the proximal promoters into five clusters based on their tissue identity (rows) and tissue specificity (columns). Thereafter, we clustered each proximal promoter using K-means clustering into five clusters. The proximal promoter of genes specific to a specific tissue tended to be mostly hypo-methylated in this tissue compared to other tissues (Figure 2a). In contrast, the 112 positively correlated genes showed the opposite pattern (Figure 2b).

### 2.4. Gene Set Enrichment Analysis for the Genes Affected by Methylation Perturbation

To identify the functional consequences underlying negatively and positively correlated genes, we performed a gene set enrichment analysis (GSEA) using EnrichR [22]. The pathway enrichment analysis with the Kyoto Encyclopedia of Genes and Genomes (KEGG) indicated the relatively high-rank of insulin- and adipocyte-related pathways for negatively correlated genes. The top 50 related pathways included “Insulin resistance” (*p* = 4.88 × 10^−4^), “Insulin signaling pathway” (*p* = 5.33 × 10^−4^), “Adipocytokine signaling pathway” (*p* = 1.075 × 10^−3^), and “peroxisome proliferator-activated receptor (PPAR) signaling pathway” (*p* = 8.83 × 10^−3^ (Table S2)). However, the pathways related to diverse biological process control and signal transduction, such as “axon guidance”, “Apoptosis”, and “neural crest cell development”, were enriched in the positively correlated gene set.

**Figure 3:**
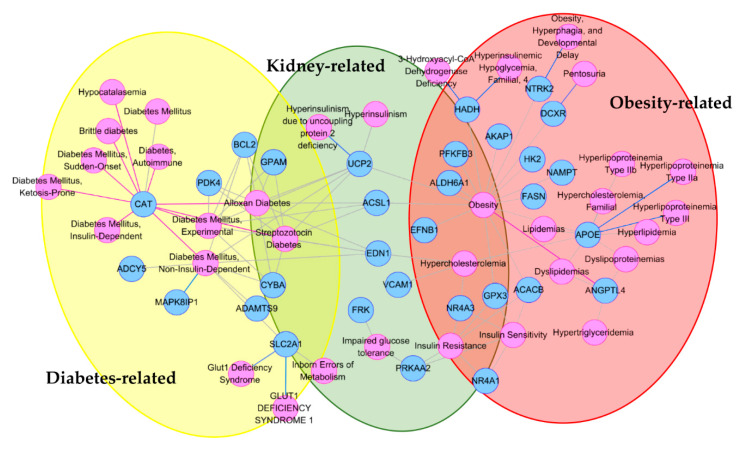
The gene–disease network for metabolic diseases. Pink nodes indicate disease names. Blue nodes represent the subsets of genes identified in our results. Red, yellow, and green circles correspond to obesity-, diabetes-, and kidney-related diseases, respectively.

We next investigated the disease association of the negatively correlated genes using the gene–disease association network (DisGeNET) [23] in EnrichR and the Cytoscape plugin [24] (Figure 3). Disease enrichment analysis showed diabetes- and obesity-related diseases to be high-ranked. The top 50 diseases included “Obesity” (*p* = 1.24 × 10^−13^), “Diabetes” (*p* = 2.68 × 10^−10^), “Diabetes Mellitus, Non-Insulin-Dependent” (*p* = 3.79 × 10^−10^), and “Diabetes Mellitus” (*p* = 6.43 × 10^−9^ (Table S3)).

We also analyzed the chromatin status to confirm that the positively correlated genes contributed to metabolic diseases. Approximately, 21% (23/112) of positively correlated genes were actively transcribed in the respective tissue (the red-colored rectangles from the fourth to eighth column in Figure S4).

### 2.5. Validation of the Epigenetic Markers Associated With Obesity

**Figure 4:**
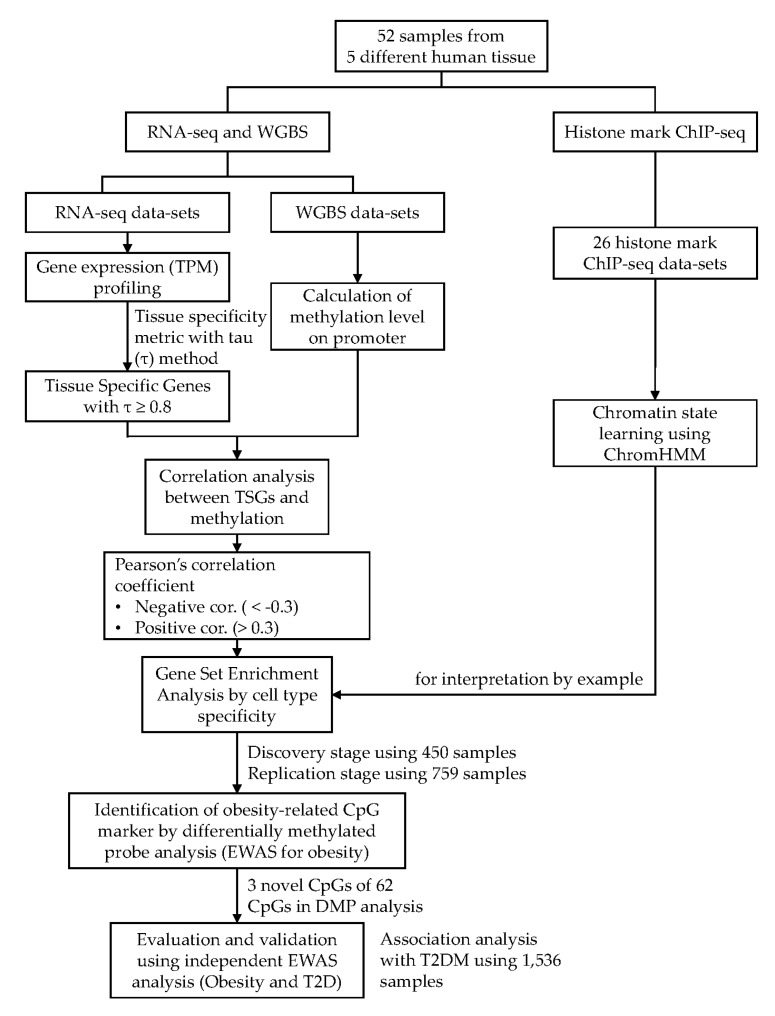
Schematic workflow.

To confirm whether the TSG promoter methylation markers are associated with diseases, we conducted epigenomic association analyses, adjusted for age and gender, between differentially methylated probes (DMPs) on adipose-specific genes and obesity using 200 obese and 250 controls (Figure 4). A recent functional study suggested that the methylation of the first intron has both a positive and negative correlation with gene expression [25]. Thus, we expanded each range of the promoter regions from 2000 base pairs upstream to the first intron. In total, 4443 CpGs located in the expanded promoter region of adipocyte-specific genes were selected for the association analyses. Of the 4443 CpGs, 62 satisfied the significance threshold with the Bonferroni correction (*p* = 1.13 × 10^−5^, 0.05/4,443) in the discovery stage. Of the 62 CpGs, three CpGs were newly identified, and these satisfied the statistical significance criteria with a nominal *p*-value of 0.05 in the subsequent replication stage, using 759 independent data (Table 2, and Table S4). We further performed an annotation analysis of the epigenome-wide association study (EWAS (EWAS catalog and EWAS Atlas)) and the GWAS (GWAS catalog within ± 100 kb of CpGs). The EWAS annotation results showed that a CpG (cg27589809) was previously reported in lipid-related traits such as triglycerides, high-density lipoproteins, and the total cholesterol. Moreover, three CpGs were colocalized with the GWAS risk loci associated with obesity-related traits or metabolite levels within ± 100 kb (Table 2).

We performed epigenomic association analyses, adjusted for age, gender, and body mass index BMI, between four CpGs and Type 2 Diabetes (T2D) using 1534 data (Table 2). Interestingly, cg17075888 on the *PDK4* gene also showed statistical significance for T2D (*p* = 2.30 × 10^−13^). In addition, a re-analysis of the *PDK4* gene expression on omental adipose tissue using the Gene Expression Omnibus (GEO) expression data set (GDS3688) showed that the *PDK4* gene expression of obese was higher than that of controls (Figure S5).

## 3. Discussion

In this study, we aimed to address whether the differences in methylation in the promoter region affect the expression of the tissue-specific genes potentially associated with diseases.

We profiled the DNA methylation and gene expression of five different types of tissues using matched samples, and investigated the TSGs affected by DNA methylation perturbation. Excluding the non-TSGs that were expressed either high or low across tissues (lower cluster in Figure 1a), we identified 677 genes (TSGs) specifically expressed in their respective tissues (upper cluster in Figure 1a). Concerning the GTEx, we identified the shared genes expressed in a specific tissue. Approximately 20% (160/677) were shared by both the data sets (Figure 1d); this percentage might reflect the differences in the data size between our set (*n* = 52) and the GTEx (*n* = 1,997). Of the 677 TSGs, 449 were affected by methylation perturbation in the proximal promoter and showed a dominant, negative regulation; approximately 75% (337 of 449) of the TSGs were negatively correlated, whereas 25% (112 of 449) were positively correlated with the proximal promoter methylation pattern (Table 1 and Table S5). This regulation pattern supported the findings of a recent cancer genome study [26]. Figure 2 shows a heatmap of the proximal promoter methylation patterns for 337 negative (Figure 2a) and 112 positive correlations (Figure 2b) of TSGs using supervised hierarchical clustering.

To explore the functional role of TSGs, we conducted a GSEA. The TSGs that negatively correlated with the promoter methylation were mostly enriched in metabolic diseases, such as diabetes, obesity-related pathways, or ontologies (Table S2). For example, *FASN* is a gene encoding for a fatty acid synthase and is known to play a key role in the regulation of obesity [27]; an increased *FASN* expression was associated with an impaired insulin sensitivity [28]. *FASN* might also play an important role in the development of obesity-related T2D [29]. The gene–disease network in DisGeNET showed the TSGs negatively regulated by promoter methylation to be tightly interconnected, and their roles to be distinctly enriched in T2D, obesity, and kidney function (Figure 3). Particularly, hub genes, such as *UCP2*, *CAT*, and *APOE*, having ≥ 6 edges (diseases) in the network, showed an interesting functional impact. Of note, *UCP2* is reportedly associated with insulin secretion, fatty acid metabolism, and glucose sensing [30,31,32].

Contrary to negative correlation, most TSGs that positively correlated with promoter methylation were not functionally enriched in the GSEA, although some of them, such as *IGFBP2*, had associations with diabetes, insulin resistance, insulin sensitivity, and obesity [33]. We speculated that the gene set might have been too small to identify a functional enrichment. Based on our results, we suggested the possibility that positively correlated genes are associated with chromatin states and alternative epigenetic regulatory mechanisms. Figure S4 represents the specific tissues and chromatin states of five tissues for each positively correlated gene. Most of the red color matching the tissue of the third column indicated the promoter of each gene to be an active promoter. According to the state annotation of the “Expanded 18-state model” in the Roadmap Epigenomics Project [34], red-related colors indicated active TSSs, flanking TSSs, and flanking TSS upstream states. These active states indicated enriched H3K4me3 and H3K27ac histone marks. Since H3K4me3 and H3K27ac are tightly related to chromatin openness [35], the existence of these marks implies that the region is being actively transcribed. Moreover, a previous study had suggested that the establishment of DNA methylation during early development is possibly mediated by histone H3K4me3 [36].

We confirmed that the promoter CpG status of TSG controlling gene expression is highly associated with metabolic diseases. From the epigenetic analysis using approximately 2000 participants, we identified 62 obesity-related DMPs. The disease association of 3, out of 62, DMPs was newly identified in the current study. Although there was no direct link with the EWAS, most of the genes with three embedded DMPs colocalized with the GWAS risk locus, suggesting a potential functional relevance, or a direct functional evidence related to obesity or obesity risk factors (Table 2).

A novel methylation marker, cg17075888 (*PDK4*) also seemed intriguing, since this gene was extensively researched for potential therapeutic targets; a *PDK4* inhibitor has been reported as a potential drug target for metabolic diseases, such as T2D [37]. Pyruvate dehydrogenase kinase 4, encoded by *PDK4*, is an enzyme located in the matrix of the mitochondria, which catalyzes the oxidative decarboxylation of pyruvate to form acetyl-CoA [38,39]. Therefore, *PDK4* plays a key role in fatty acid metabolism, glucose metabolism, and the tricarboxylic acid (TCA) cycle [39,40,41]. Changes in the activity of the *PDK* family, including *PDK4*, mediate the inhibitory effect of fatty acids on glucose metabolism; thus, *PDK4* plays a vital role in obesity and metabolic diseases [42]. *PDK4* is also reported as one of the primary target genes of peroxisome proliferator-activated receptor γ (*PPARγ*) [43,44]. *PPARγ* is mainly located in adipose tissue and is involved in various functions such as adipocyte differentiation, lipid metabolism, insulin sensitivity, and fatty acid storage [44,45,46]. There is additional information on the relationship between *PDK4* and obesity. For example, the GEO expression data set (GDS3688) showed that the *PDK4* gene expression of obese was higher than that of controls (Figure S5). Several studies regarding the promoter methylation of *PDK4* have indicated the *PDK4* methylation levels to negatively correlate with BMI (body mass index) in skeletal muscle samples, and weight loss to be associated with methylation changes of *PDK4* promoters [47,48,49,50,51,52]. Furthermore, an intergenic variant, rs6465468 nearby the *ASB4* gene associated with BMI, is colocalized with *PDK4* (about 43.5 kb downstream of *PDK4* (Table 2 and Figure S6a)) [53]. However, to date, no previous study has identified which of the obesity-associated methylation markers directly affected the target gene expression. This study firstly suggests that a negative correlation between the hypo-methylated CpG (cg17075888) on the *PDK4* gene and its expression was highly associated with obesity.

Other novel methylation markers, cg27589809 (*CISH*), and cg20560869 (*NR4A1*), are associated with obesity. Cytokine inducible *SH2* containing protein, encoded by *CISH*, is a suppressor in the JAK/STAT pathway and is known to be involved in the regulation of activity of multiple important cytokines, including insulin, leptin, growth hormone, IL-6, prolactin, and interferons. In particular, leptin plays an important role in obesity-related factors, such as body fat storage, and the regulation of body weight [32]. A recent epigenome-wide association study (EWAS) has reported CpGs (cg23005227 and cg21585138) associated with *CISH* in African-Americans (Figure S6b) [42]. These two CpGs were located in exon 3 of *CISH* that were in 10 kb downstream of cg27589809 [54], suggesting that cg27589809 has a more causal effect on the regulation of the target gene expression than those of previously reported two markers. Nuclear receptor subfamily 4 group A member 1, encoded by *NR4A1*, is known to be a mediator of fasting-induced *PPARγ2* regulation in white adipose tissue (WAT) [55]. *NR4A1* is reported to have an association with chronic low-grade inflammation [56]. Obesity is associated with chronic low-grade inflammation, which results in insulin resistance, T2D, vascular disease, chronic renal failure, several cancers, and endocrine and behavioral abnormalities [57].

Considering that we only assessed the tissue specificity of 52 samples of 5 tissues, in future studies, more extensive data sets are required to enhance the statistical power of the analyses. As mentioned above, a relatively small-sized data set limited TSG detection, since the assessment of tissue specificity is fundamentally calculated based on the averaging expression value of tissue samples. To assess the tissue specificity accurately, it would be important to ensure both the tissue diversity and sample size. As one of the first integrative analyses of whole genome-scale DNA methylation and RNA sequencing from matched samples of human tissues [15,34,58], this study could also provide additional evidence of the metabolic disease-related gene–disease network and the EWAS results, considering the epigenetic regulation of gene expression.

## 4. Materials and Methods

### 4.1. Analysis Scheme

We generated WGBS, mRNA-seq, and histone modification ChIP-seq data from 9 pancreatic islets, 21 adipose tissues, 10 kidney tissues, 8 SMCs (skeletal muscle cells), and 4 fibroblasts isolated from humans (Tables S6 and S7). First, we profiled the gene expression from the RNA-seq data sets; the tissue specificity of each gene was evaluated by the Tau method [59]. We then defined the genes with a Tau value greater than a certain threshold as TSGs. Thereafter, we calculated the methylation level of each promoter region (from 200 base pairs upstream to 50 base pairs downstream of the TSS (transcription start site)) using WGBS data sets. We next conducted a correlation analysis between the gene expression levels and promoter methylation for each gene. Each gene was classified according to the tendency of Pearson’s correlation coefficient and by the tissue specificity. Genes of each group were subjected to an enrichment analysis using Enrich to predict the gene ontology, relationship with a disease, and biological pathways associated with chronic diseases. Besides, DisGeNET, a database of gene–disease associations, was analyzed to re-construct the disease and gene networks. Finally, we identified obesity-associated CpGs through an obesity EWAS analysis using about 2000 DNA methylation samples (Table S8). The analysis scheme of this study is illustrated in Figure 4.

### 4.2. Study Samples

Islet, adipocyte, and kidney samples were obtained from Seoul Asan Medical Center and Seoul National University Hospital. All tissues were collected from patients of pancreatectomy, obesity, or nephrectomy. The isolation of each tissue was performed according to the traditional cell isolation protocol, with some modification, depending on the tissue. In brief, the traditional protocol of mincing and digesting the tissues was performed, followed by a centrifugation to collect the cells [60]. Islets were digested by liberase or collagenase. After centrifugation, the adipocytes were isolated from the supernatant, whereas the kidney cells were present in the pellet. The kidney cells were separated into mesangial cells and podocytes using magnetic-activated cell sorting (MACS). All samples were aliquoted into three vials: two were reserved for DNA or RNA extraction, and one for a chromatin-fixation with formaldehyde for next-generation sequencing.

DNA samples for the Infinium EPIC array (Illumina, CA, USA) were recruited from the Health Examinees Study (HEXA). We conducted the association analysis using 450 samples (200 obese, BMI: 31.6 ± 1.6 kg/m^2^ and 250 control, BMI: 21.4 ± 1.1 kg/m^2^) in this cohort.

### 4.3. Ethics Approval and Consent to Participate

All of the islet, adipocyte, and kidney samples were collected after obtaining informed consent from the participants, in accordance with national laws and institutional ethical requirements. All procedures were approved by the Institutional Review Boards (IRB) of Seoul Asan Medical Center (S2013-0170-0008, approved on 6 August 2014) and Seoul National University Hospital (H-1302-095-466, approved on 19 June 2014).

Fibroblasts and skeletal muscle cells were obtained from the Wonkwang University Biobank of Korea, with written informed consent, under the Korea National Institute for Bioethics Policy (KoNIBP) and the corporation’s IRB approval (P01-201606-31-001, approved on 7 June 2016).

All DNA samples for the DNA methylation array were from the National Biobank of Korea, and were obtained with written informed consent, following the KoNIBP and corporation’s IRB (P01-201703-31-004, approved on 13 March 2017 and LASIRB-20180222-001/002, approved on 22 February 2018).

This study was approved by the institutional review board at the Korea National Institute of Health (2017-03-02-R-A, approved on 5 September 2017).

### 4.4. Processing of Sequencing Data

Poly A-captured mRNA libraries were sequenced on a HiSeq 2500 platform (Illumina, San Diego, CA, USA) with 100-bp paired-end reads. Raw sequencing data were trimmed off adaptor sequences by Trimmomatic (ver. 0.36) [61]. All trimmed reads were mapped to the GRCh37/hg19 reference genome using open-source software, STAR (version 2.5.3a, https://github.com/alexdobin/STAR, 18 Mar 2017) [62] in accordance with the ENCODE RNA-seq for long RNAs [58]. The expression levels from the aligned RNA-seq data were estimated by open-source software, RSEM (version 1.3.0, https://github.com/deweylab/RSEM, 10 Dec 2017) [63] based on the GENCODE release 19 gene annotation. Genes that expressed transcripts per million (TPM) > 0 in at least 1 of the samples were selected.

The sodium bisulfite converted DNA libraries were sequenced by the Illumina HiSeq 2500 and HiSeq X Ten systems, with 100-bp paired-end reads. Raw reads were trimmed off adapter sequences using Trimmomatic (ver. 0.36). All trimmed reads were aligned with the GRCh37/hg19 human reference genome using BSMAP (ver. 2.87) [64], with the option for reporting the unique mapped read. Duplicated reads were discarded using Picard (ver. 2.5.0) [65]. Methylation calling was conducted using Python script in the BSMAP tool for a sequencing depth of 10 or more.

Histone modification libraries were sequenced in an Illumina HiSeq 2500 system, with 100-bp paired-end reads. For each sample, 6 histone modification marks and inputs (control) were sequenced. All ChIP-seq reads were trimmed by Trimmomatic and mapped to the GRCh37/hg19 human reference genome using the Burrows-Wheeler Alignment Tool with maximal exact matches (BWA-MEM) algorithm on a BWA mapper (ver. 0.7.15-r1140) [66] with the default setting. Unmapped and duplicated reads were marked and eliminated by Picard and SAMtools (ver. 0.1.19-96b5f2294a) [67]. To investigate the chromatin states for the promoter region of the histone modification samples, we conducted a ChromHMM (ver. 1.14) analysis [68,69]. Then, we assigned chromatin states of the samples using the “Expanded 18-state model” generated by Roadmap Epigenomic Consortium [34].

For the batch adjustment, we used the ComBat method of sva package in R [70,71].

### 4.5. Assessment of Tissue Specificity

We used τ (Tau) as a measure of the tissue specificity [59]. For all genes with a TPM > 1, the mean TPM of each of the five tissues was applied to the Tau formula:

$$\tau =\frac{\sum_{i=1}^{n}\left(1-\hat{x_{i}}\right)}{n-1};\;\hat{x_{i}}=\frac{x_{i}}{\underset{1\leq i\leq n}{\max}x_{i}}$$

Tau ranges from 0 (expressed ubiquitously) to 1 (tissue-specific). We calculated the Tau values from the TPM of each gene in the sample and discarded those with a TPM of less than 1 in all samples. A recent study has shown Tau to be the best metric, among 9 methods, for measuring tissue specificity [59]. We finally selected the genes with Tau ≥ 0.8, and the highest expressed gene in a certain tissue was assigned as specific to that tissue (Pseudo-code in Supplementary Notes).

### 4.6. Correlation Analysis

We performed two types of correlation analyses. First, we investigated whether each assay showed tissue specificity, using a pairwise correlation test. For each expression and methylation matrix, the pairwise correlation coefficients across all samples were calculated by the cor function in R [72], using a log-transformed TPM value and the mean methylation level, respectively. A correlation plot was generated using the corrplot function of the corrplot package [73]. We then performed a correlation analysis to identify the strength of the relationship between the gene expression and promoter methylation level of the proximal promoter for each gene. We calculated the Pearson’s correlation coefficients using the cor function in R.

### 4.7. Clustering Heatmap Analysis

We conducted clustering and constructed heat maps using the ComplexHeatmap package [74] in R. The clustering distance used the Pearson method and all other options used default values. We used log-transformed TPM values for Figure 1a, and the methylation value scaled by row (gene) for Figure 2, to draw the heat map.

### 4.8. Functional Annotation and Visualization

For each gene, positively or negatively correlated with the promoter methylation, we performed a KEGG pathway analysis, DisGeNET, and gene ontology enrichment test on EnrichR (http://amp.pharm.mssm.edu/Enrichr). We downloaded *p*-value-sorted text files and then extracted the top signals.

We conducted a network analysis for the negatively correlated genes using the DisGeNET Cytoscape Plugin on Cytoscape [75]. We ran the tool under the options “CURATED” for source, “ANY” for association type, “Nutritional and Metabolic Diseases”, and disease class.

### 4.9. Identification of Obesity-Related CpG Marker

We conducted a differentially methylated probe (DMP) analysis for 168 adipocyte-specific genes in the HEXA cohort, as an independent cohort. In the DMP analysis for CpGs in the promoter region (extended from 2 kb upstream of the TSS to the first intron) of adipocyte-specific genes, we identified DMPs satisfying the Bonferroni corrected *p*-value (*p* = 1.13 × 10^−5^). The collected DMPs were validated using another independent data set (of 759) as a replication set. Finally, to examine the relationship between these DMPs and T2D, we conducted a T2D association test using 1536 data sets with an adjusted BMI. We further confirmed whether the DMPs were reported as obesity-related traits in the EWAS catalog and EWAS Atlas, and also checked the disease-associated SNPs around these DMPs in the GWAS catalog. Next, we conducted an in silico validation using the Gene Expression Omnibus (GEO) expression data set on omental adipose tissue (GDS3688) [76].

### 4.10. Data Availability

The sequencing data analyzed during the current study are available in the repository: the European Genome-phenome Archive (EGA) database (https://www.ebi.ac.uk/ega/home) under the study accession number EGAS00001001774. In addition, the processed bigwig data are available in the IHEC data portal (https://epigenomesportal.ca/ihec/).

## 5. Conclusions

In this study, we analyzed the differences in the correlation tendencies between the gene expression and promoter methylation related to metabolic diseases. Correlation analysis between the promoter methylation and gene expression in different tissue types revealed a majority of metabolic diseases-related TSGs, including T2D and obesity. With the additional confirmation of the epigenetic changes in the EWAS, we comprehensively suggested novel epigenetic regulatory features of a potential drug target for T2D, namely *PDK4*.

## Figures and Tables

**Table 1:** Number of correlated genes in five different tissues.

Tissue	Negatively Correlated Genes	Positively Correlated Genes
Adipocyte	162	19
Fibroblast	19	16
Islet	51	14
Kidney	91	49
SMC ^1^	14	14
Total	337	112

^1^ Skeletal muscle cell.

**Table 2:** Obesity-associated methylation markers newly identified in the current study.

CpG ID	Chrom-osome (hg19)	Position	HGNC Symbol	Discovery (*n* = 450) *p*-Value	Replication (*n* = 759) *p*-Value	Replication (*n* = 759) *p*-Value (adj. T2D)	T2DM (*n* = 1534) *p*-Value (adj. BMI)	EWAS Catalog ^1^ (*p* < 0.05)	EWAS Atlas ^2^ (*p* < 0.05)	GWAS Catalog ^3^ (Disease Associated SNP within ± 100 kb at the CpG Site)
cg27589809 *	3	50650410	*CISH*	1.69 × 10^−8^	1.61 × 10^−2^	1.48 × 10^−2^	8.31 × 10^−1^	Triglycerides, Phospholipids to total lipids ratio, High-density lipoprotein cholesterol, Age, smoking, HIV infection	smoking	Waist-to-hip ratio adjusted for BMI, Height, Eosinophil counts
cg17075888 *	7	95225339	*PDK4*	4.75 × 10^−6^	1.10 × 10^−8^	1.02 × 10^−4^	2.30 × 10^−13^			Body Mass Index (rs6465468, reported gene: *ASB4*), Metabolite levels
cg20560869	12	52447054	*NR4A1*	1.44 × 10^−6^	3.45 × 10^−3^	1.56 × 10^−2^	5.89 × 10^−1^			Metabolite levels, Lung function, neutrophil eosinophil counts, Red blood cell count, Mean corpuscular hemoglobin, Interleukin-13 levels

^1^ EWAS Catalog (http://www.ewascatalog.org/), ^2^ EWAS Atlas (https://bigd.big.ac.cn/ewas), ^3^ GWAS Catalog (https://www.ebi.ac.uk/gwas/). * These CpG markers are shown in Figure S6.

## Supplementary Materials

Supplementary materials can be found at https://www.mdpi.com/1422-0067/21/14/5056/s1. Table S1. Six-hundred and seventy-seven tissue-specific genes; Table S2. Kyoto Encyclopedia of Genes and Genomes (KEGG) pathway in EnrichR (gene set enrichment analysis (GSEA [*p* < 0.05])); Table S3. Top 100 gene–disease association network (DisGeNet) diseases in EnrichR (GSEA); Table S4. Replication of obesity-associated methylation markers newly identified in the current study; Table S5. Four-hundred and fourty-nine tissue-specific genes affected by proximal promoter methylation; Table S6. Sample information (number of mapped reads); Table S7. Characteristics of 52 subjects for sequencing data; Table S8. General characteristics of the subjects for an analysis on obesity-related differentially methylated CpGs (* body mass inded (BMI) > 30 kg/m^2^, † BMI > 27 kg/m^2^). Figure S1. Adjustment of batch effects. (a) PCA plot of 52 RNA-seq data after batch correction. (b) PCA plot of 52 whole genome bisulfite sequencing (WGBS) data after batch correction. (c) Distribution of log-transformed transcripts per million (TPM) value of RNA-seq data; Figure S2. Pairwise correlation analysis of RNA-seq. This analysis was performed using 11,111 genes with a TPM of at least 1 of all the samples of the protein code based on the GENCODE v19 gene model. Samples of intra-tissue revealed a high correlation, but samples of inter-tissue revealed weak correlations; Figure S3. Comparing typical and proximal promoters, where 2000 bp stands for the region from 2000 bp upstream of the transcription state site (TSS) to TSS, 250 bp stands for the region from 250 bp upstream of the TSS to TSS; Figure S4. Chromatin states for positively correlated genes with proximal promoter methylation. Red-, green-, yellow-, and grey-related colored states represent an active transcription, elongation, enhancer, and repressive transcription states, respectively; Figure S5. *PDK4* expression of a geo data set (GDS3688); Figure S6. Examples of obesity-associated methylation markers and related genes. The identified markers were obesity-associated markers that we identified. (a) cg17075888, located in the promoter of *PDK4*, (b) cg27589809, located in the promoter of cg27589809 (*CISH*).

## Abbreviations

TSG	Tissue-specific gene
WGBS	Whole Genome Bisulfite Sequencing
ChIP-seq	Chromatin Immunoprecipitation Followed by Sequencing
IHEC	International Human Epigenome Consortium
TPM	Transcripts per Million
GTEx	The Genotype-Tissue Expression
GSEA	Gene Set Enrichment Analysis
KEGG	Kyoto Encyclopedia of Genes and Genomes
TSS	Transcription Start Site
TFBS	Transcription Factor Binding Site
DisGeNET	Gene–disease Association Network
CKD	Chronic Kidney Disease
