# Supplementary material for: Integrated Analysis of Tissue-Specific Promoter Methylation and Gene Expression Profile in Complex Diseases
Source: Int J Mol Sci. 2020 Jul 17;21(14):5056. doi: 10.3390/ijms21145056 (PMC7404266; doi:10.3390/ijms21145056)
Supplement: Supplementary file 1 [file ijms-21-05056-s001.zip › Supplementary Figures and Notes.pdf]

Supplementary Figures

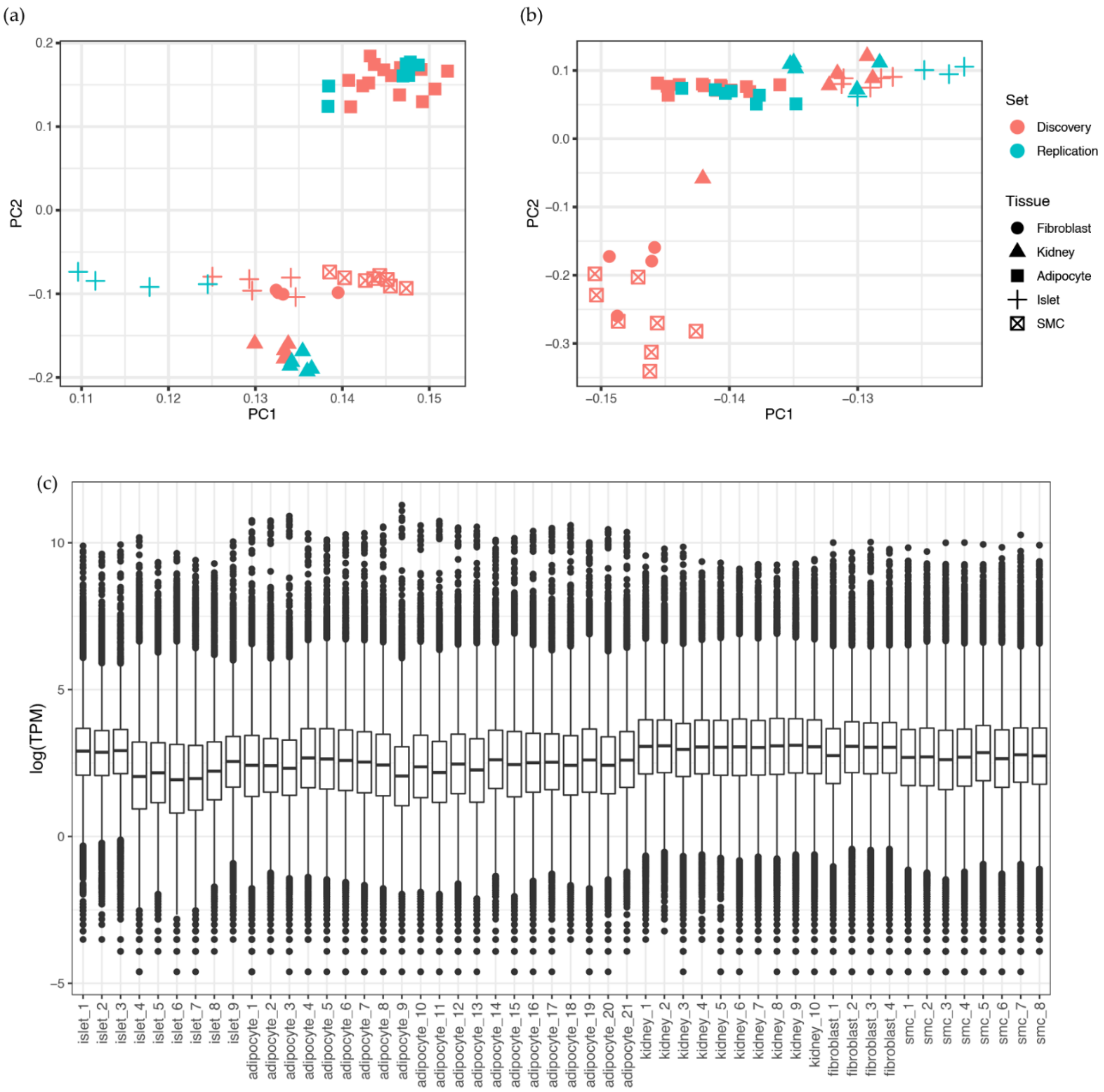

Figure S1. Adjustment of batch effects. (a) PCA plot of 52 RNA-seq data after batch correction. (b) PCA plot of 52 WGBS data after batch correction. (c) Distribution of log-transformed TPM value of RNA-seq data.

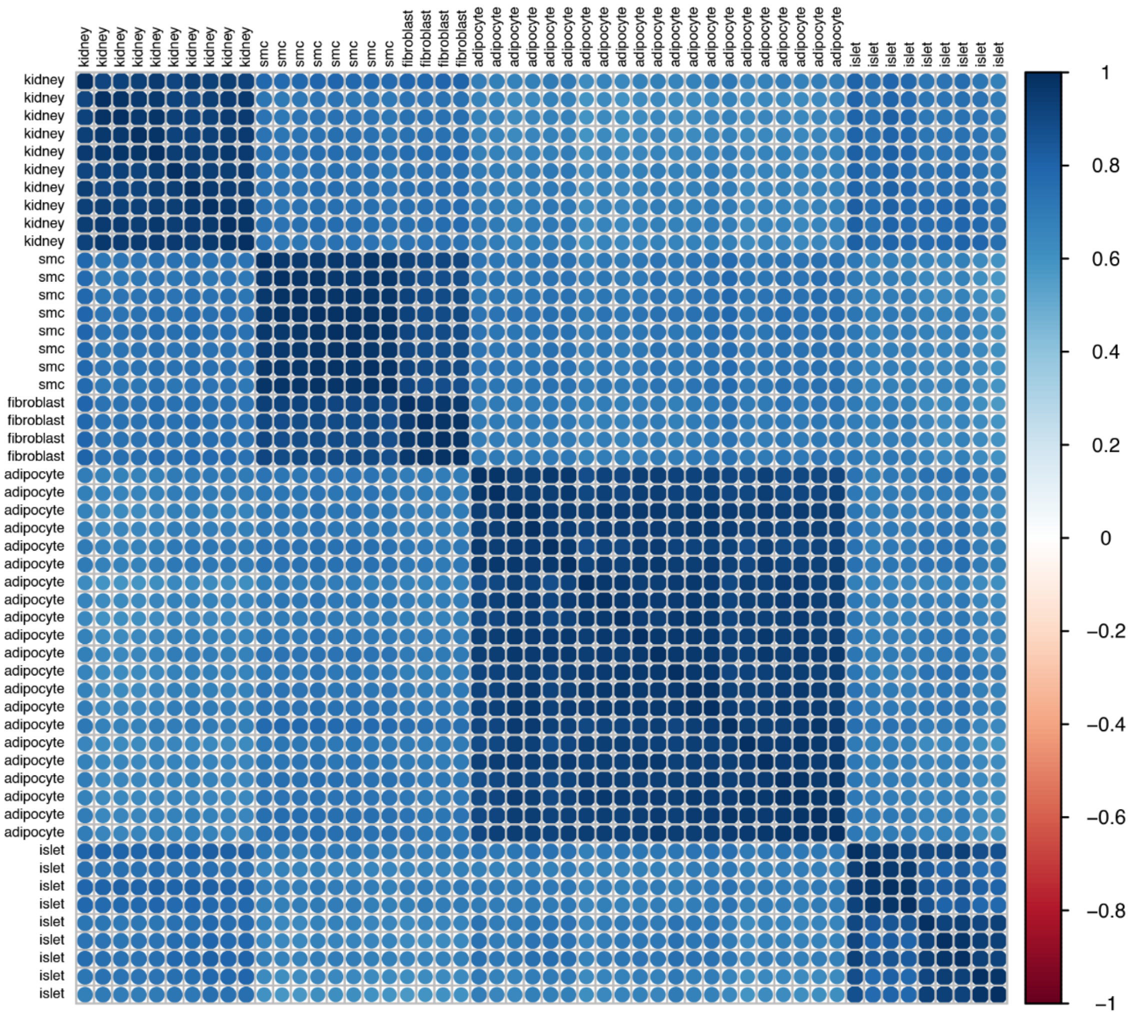

Figure S2. Pairwise correlation analysis of RNA-seq. This analysis was performed using 11,111 genes with a TPM of at least 1 of all the samples of the protein code based on the GENCODE v19 gene model. Samples of intra-tissue revealed highly correlated, but samples of inter-tissue revealed weak correlations.

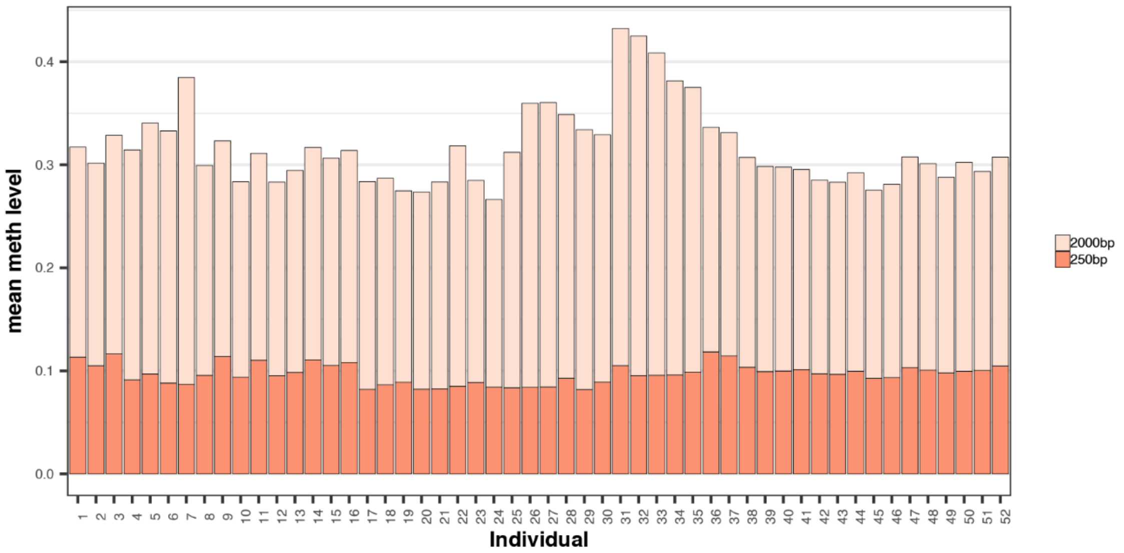

Figure S3. Comparing typical and proximal promoters, where 2000 bp stands for the region from 2000 bp upstream of TSS to TSS, 250 bp stands for the region from 250 bp upstream of TSS to TSS.

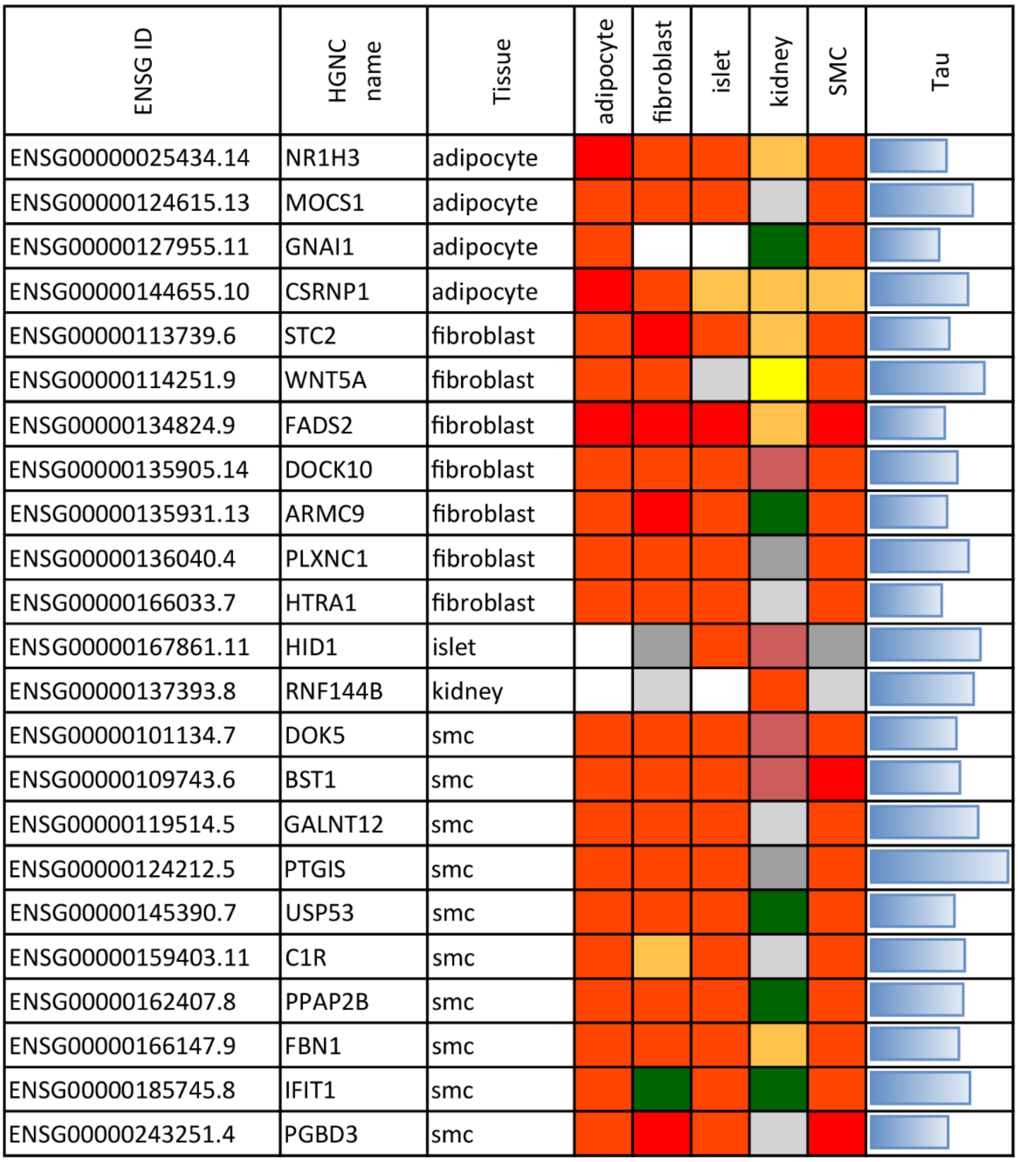

Figure S4. Chromatin states for positively correlated genes with proximal promoter methylation. Red-, green-, yellow-, and grey-related colored states represent active transcription, elongation, enhancer, and repressive transcription states, respectively.

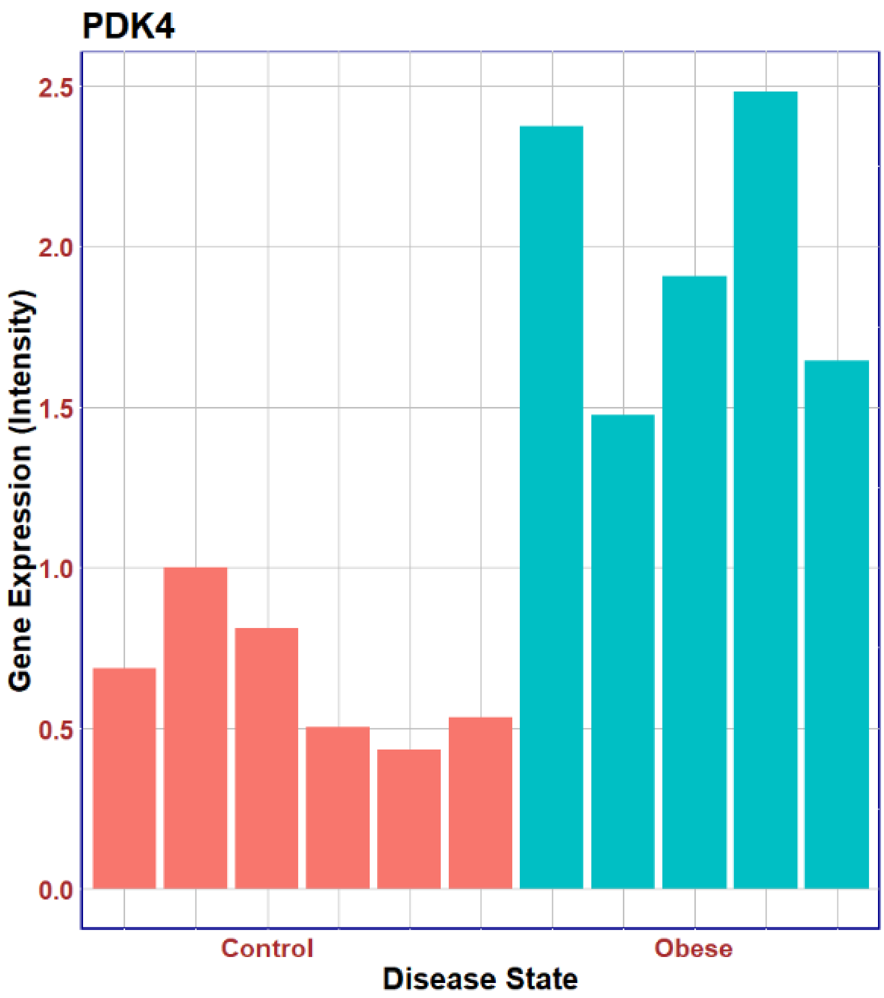

Figure S5. PDK4 expression of a geo data set (GDS3688).

(a)

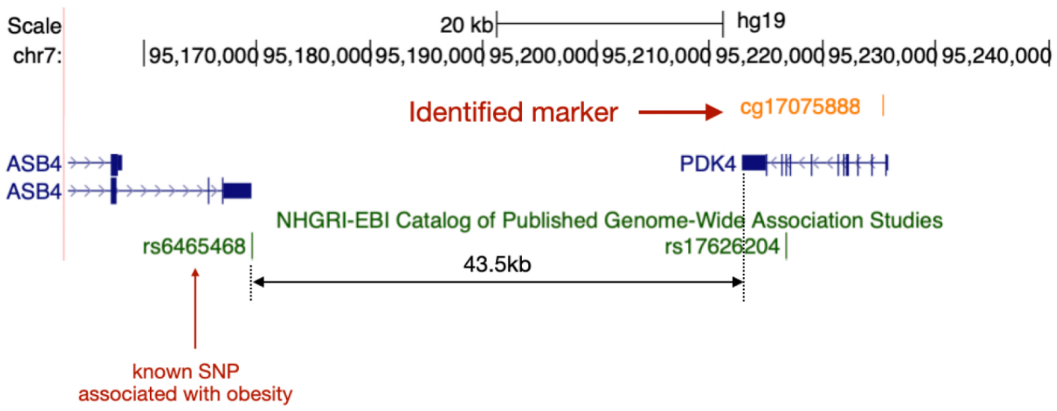

(b)

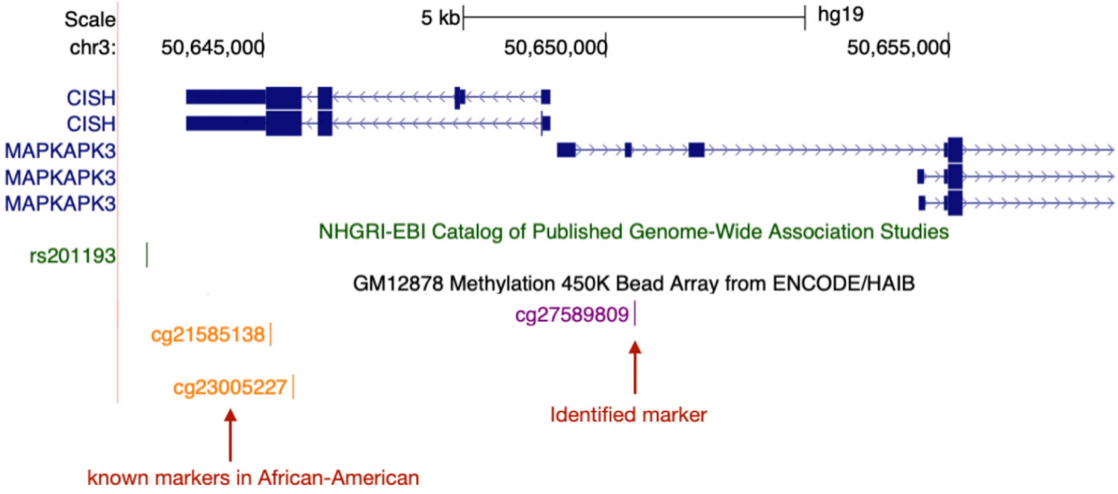

Figure S6. Examples of obesity-associated methylation markers and related genes. The identified markers were obesity-associated markers that we identified. (a) cg17075888, located in the promoter of PDK4, (b) cg27589809, located in the promoter of CISH.

Supplementary Notes

The pseudo-code for calculation tissue specificity using tau method.

From the TPM matrix of 36 RNA-seq data (discovery set) aligned by STAR and RSEM, a TPM matrix of the entire sample was created using bash script. Then, the formula of the tau method that mentioned in the Method section was implemented directly in the R environment to evaluate the tissue specificity of each gene. The pseudo-code of tau method is below:

Avg.matrix by tissue ← Average TPM of each gene by tissue.

Avg.dataframe ← cbind(all avg.matrix by tissue)

Max.tissue ← Avg.TPM of max tissue

Sum.matrix ← Sum of all samples' TPM by gene

TSI ← Max.tissue divided by Sum.matrix by gene

Tau ← 1 / (total number of tissues -1) * (total number of tissues – (1 / TSI))

The pseudo-code for correlation analysis between gene expression and promoter methylation

We calculated average methylation level of each promoter region using in-house script using python and generated a matrix of methylation level for the entire sample in the same way as TPM matrix of the entire sample. Then, the correlation analysis was performed in R. The pseudo-code is below:

```
for each gene in TPM and methylation matrix

cor(TPM of each gene, methylation level of each gene)
```
