# Supplementary material for: Integrated Analysis of Tissue-Specific Promoter Methylation and Gene Expression Profile in Complex Diseases
Source: Int J Mol Sci. 2020 Jul 17;21(14):5056. doi: 10.3390/ijms21145056 (PMC7404266; doi:10.3390/ijms21145056)
Supplement: Supplementary file 1 [file ijms-21-05056-s001.zip › Table Legends.pdf]

Table Legends

Table S1. 677 Tissue-specific genes

Table S2. KEGG pathway in EnrichR (GSEA) ($p < 0.05$)

Table S3. Top 100 DisGeNet Diseases in EnrichR (GSEA)

Table S4. Replication of obesity-associated methylation markers newly identified in the current study

Table S5. 449 Tissue-Specific Genes affected by proximal promoter methylation

Table S6. Sample information (Number of mapped reads)

Table S7. Characteristics of 52 subjects for sequencing data

Table S8. General characteristics of the subjects for analysis on obesity-related differentially methylated CpGs (* BMI > 30 kg/m^2, † BMI > 27 kg/m^2)
